# 初诊骨髓增生异常综合征伴原始细胞增多患者治疗前后基因突变状态与去甲基化药物治疗疗效及生存关系的研究

**DOI:** 10.3760/cma.j.cn121090-20241210-00553

**Published:** 2025-05

**Authors:** 婷 钟, 铁军 秦, 泽锋 徐, 丽娟 潘, 士强 曲, 蒙 焦, 清妍 高, 志坚 肖, 冰 李

**Affiliations:** 1 中国医学科学院血液病医院（中国医学科学院血液学研究所），血液与健康全国重点实验室，国家血液系统疾病临床医学研究中心，细胞生态海河实验室，天津 300020 State Key Laboratory of Experimental Hematology, National Clinical Research Center for Blood Diseases, Haihe Laboratory of Cell Ecosystem, Institute of Hematology & Blood Diseases Hospital, Chinese Academy of Medical Sciences & Peking Union Medical College, Tianjin 300020, China; 2 天津医学健康研究院，天津 301600 Tianjin Institutes of Health Science, Tianjin 301600, China

**Keywords:** 骨髓增生异常综合征, 去甲基化药物, 主克隆, 疗效, 生存, Myelodysplastic syndromes, Hypomethylating agents, Major clones, Efficacy, Overall survival

## Abstract

**目的:**

探索接受去甲基化药物（HMA）单药治疗的骨髓增生异常综合征伴原始细胞增多（MDS-EB）患者治疗前后基因突变状态与疗效及预后的关系。

**方法:**

2016年6月至2023年9月于中国医学科学院血液病医院完成了至少4个疗程HMA单药治疗且治疗前进行了二代测序（NGS）的69例初治MDS-EB患者，回顾性分析其临床特征、疗效、生存时间以及生存情况。比较HMA治疗前后基因突变症状对疗效与预后的影响。

**结果:**

①69例患者中，男47例，女22例，中位年龄62（41～80）岁。MDS-EB1 39例，MDS-EB2 30例，中位治疗6（4～35）个疗程。中位随访22（5～72）个月，中位生存期32（95％*CI*：27～43）个月。②HMA治疗前是否伴有DTA（DNMT3A、TET2和ASXL1）突变、信号转导基因突变、转录因子突变或剪接因子突变与4个疗程内最佳疗效、疗效持续时间（DOR）和总生存（OS）均无显著相关性。TP53突变状态与DOR相关且OS时间显著缩短，伴有双打击TP53突变者、单打击TP53突变者和非TP53突变者的中位DOR分别为3（95％*CI*：1～10）、10（95％*CI*：3～34）和16（95％*CI*：8～27）个月（*P*＝0.032），中位OS时间分别为16（95％*CI*：7～38）、15（95％*CI*：6～40）和35（95％*CI*：14～91）个月（*P*<0.001）。③修订的国际预后积分系统（IPSS-R）和含分子遗传学指标的国际预后积分系统（IPSS-M）均不能预测接受HMA治疗4个疗程内最佳疗效和DOR。④非TP53突变患者，主克隆显著清除组（14例）和主克隆非显著清除组（10例）中位OS时间分别为55（95％*CI*：9～106）个月和31（95％*CI*：16～184）个月（*P*＝0.013）。HMA治疗有效且主克隆显著清除者的3年OS率达（77.8±13.9）％。

**结论:**

MDS-EB患者接受HMA单药治疗前，单一基因突变、IPSS-R和IPSS-M预后积分系统均无法有效预测治疗效果。非TP53突变患者，通过NGS监测治疗过程中主克隆清除程度可预测接受HMA治疗的MDS患者的长期疗效。

## Abstract (en)

**Objective:**

To investigate the association between pre- and post-treatment gene mutation profiles and clinical outcomes（treatment response and prognosis）in patients with myelodysplastic syndromes with excess blasts（MDS-EB）receiving hypomethylating agent（HMA）monotherapy.

**Methods:**

The clinical characteristics, treatment efficacy, and survival outcomes of 69 treatment-naive patients with MDS-EB who underwent next-generation sequencing（NGS）before treatment and completed at least 4 cycles of HMA monotherapy at the Institute of Hematology & Blood Diseases Hospital, CAMS & PUMC, between June 2016 and September 2023, were retrospectively analyzed.

**Results:**

① The cohort comprised 47 males and 22 females with a median age of 62 years（range: 41–80）. Thirty-nine patients were classified as MDS-EB1 and 30 as MDS-EB2. The median number of treatment cycles was 6（range: 4–35）. The median follow-up duration was 22 months（range: 5–72）, and the median overall survival（OS）was 32 months（95％ *CI*: 27–43）. ② The presence of DTA（DNMT3A, TET2, or ASXL1）mutations, signaling pathway mutations, transcription factor mutations, or splicing factor mutations before HMA treatment showed no significant association with the best response within 4 treatment cycles, duration of response（DOR）, or OS. TP53 mutation status was significantly associated with DOR and shorter OS. The median DOR was 3 months（95％ *CI*: 1–10）for patients with biallelic TP53 mutations, 10 months（95％ *CI*: 3–34）for those with monoallelic TP53 mutations, and 16 months（95％ *CI*: 8–27）in patients without TP53 mutations（*P*＝0.032）. The median OS was 16 months（95％ *CI*: 7–38）, 15 months（95％ *CI*: 6–40）, and 35 months（95％ *CI*: 14–91）, respectively（*P*<0.001）. ③ Neither the Revised International Prognostic Scoring System（IPSS-R）nor the Molecular International Prognostic Scoring System（IPSS-M）could predict the best response within 4 treatment cycles or DOR in patients receiving HMA therapy. ④ Among patients without TP53 mutations, the median OS was 55 months（95％ *CI*: 9–106）for the major clone significant clearance group（*n*＝14）and 31 months（95％ *CI*: 16–184）for the major clone non-significant clearance group（*n*＝10）（*P*＝0.013）. For patients who responded to HMA treatment and had significant major clone clearance, the 3-year OS rate reached（77.8±13.9）％.

**Conclusion:**

For MDS-EB patients receiving HMA monotherapy, single gene mutations, IPSS-R, and IPSS-M could not effectively predict treatment outcomes before therapy. However, for patients without TP53 mutations, monitoring the degree of major clone clearance by NGS during treatment may predict the long-term efficacy in MDS patients receiving HMA therapy.

骨髓增生异常综合征（MDS），又称骨髓增生异常肿瘤，是一组起源于造血干细胞的异质性髓系肿瘤[1]–[2]。其中，MDS伴原始细胞增多（MDS-EB）亚型预后最差，中位生存期仅为17～23个月[3]。根据最新修订的含分子遗传学指标的国际预后积分系统（IPSS-M）[4]，43％患者属于较高危组（HR-MDS）。目前，异基因造血干细胞移植（allo-HSCT）是治愈HR-MDS唯一方法。对于不适合移植的患者，去甲基化药物（HMA）仍为此类患者的一线治疗选择[5]–[7]。目前，常规的临床指标并不能很好预测HMA疗效[8]。近年来，随着二代测序（NGS）在MDS诊断中的广泛应用，基因突变在预后评估和治疗选择中的地位日益重要，其能否预测接受HMA治疗患者的疗效及生存尚存争议。本研究我们回顾性分析了单中心69例接受至少4个疗程HMA单药治疗的初治MDS-EB患者，探索治疗前基因突变状态和治疗过程中基因突变的动态变化与疗效及预后的关系。

## 病例与方法

1. 病例资料：回顾性分析了2016年6月至2023年9月中国医学科学院血液病医院就诊且符合纳入标准的69例MDS-EB（39例MDS-EB1，30例MDS-EB2）患者的临床资料，男47例，女22例，中位年龄62（41～80）岁。纳入标准包括：①符合WHO2016诊断分型标准的初治患者；②治疗前接受了NGS基因突变检测；③接受至少4个疗程阿扎胞苷（Azacitidine，AZA）或地西他滨（Decitabine，DAC）单药治疗。

2. 染色体核型检测：骨髓细胞经过24 h培养，收集细胞常规制片，采用G或R显带技术进行核型分析，根据《人类细胞遗传学国际命名体制（ISCN）2024》进行描述，并根据修订的国际预后积分系统（IPSS-R）[9]进行染色体核型预后分组。

3. 基因突变检测：采用NGS进行基因突变检测，检测的基因包括ASXL1、SF3B1、RUNX1等141个基因。等位基因突变频率（VAF）≥2％的基因突变纳入分析，所有检测出的外显子区通过千人基因组计划、癌症中的体细胞突变目录（COSMIC）及PolymorphismPhenotyping2（PolyPhen-2）数据库筛选出致病基因。

4. 治疗方案：69例患者中，24例接受AZA单药治疗（100 mg/d×7 d），45例接受DAC单药治疗（20 mg·m^−2^·d^−1^×5 d）。上述治疗均以28 d为1个周期，直到无法耐受、疾病进展或死亡，允许因不良事件或视血细胞计数恢复情况延迟用药。5例患者在接受HMA治疗后进行了allo-HSCT。

5. 预后评估：采用IPSS-R[9]和IPSS-M[4]预后积分系统对患者进行预后评估。根据IPSS-M分组，将极低危组、低危组和中低危组归为较低危组（LR-MDS），将中高危组、高危组和极高危组归为HR-MDS。

6. 疗效评估：根据MDS国际工作组（International Working Group, IWG）于2023年修订的国际MDS疗效评估标准[10]进行评价，分为完全缓解（CR）、部分缓解（PR）、完全缓解伴有限血液学改善（CR_L_）［包括单系完全改善（CR_uni_）和两系完全改善（CR_bi_）］、完全缓解伴部分血液学改善（CRh）、血液学改善（HI）、无效（NR）和疾病进展（PD）。获得疗效定义为达到CR、PR、CR_L_、CRh或HI中的任意一种反应。总有效率（ORR）为CR、PR、CR_L_、CRh、HI率之和。疗效持续时间（DOR）为首次达到CR、PR、CR_L_、CRh或HI至疾病进展或任何原因死亡的时间。

7. HMA对主克隆清除程度：主克隆定义为患者所有的基因突变中VAF最高且≥10％的Ⅰ类非胚系突变。当多个基因突变均符合上述规定，且相互的VAF差值<10％，均视作主克隆。目前对于MDS克隆清除并无统一的定义和标准，我们参考骨髓增殖性肿瘤（MPN）分子学缓解标准[11]和IWG-2023 MDS疗效评估标准中的细胞遗传学缓解标准[10]，制定了本研究中的主克隆清除标准：HMA对主克隆的显著清除定义为治疗后VAF较治疗前下降≥50％；HMA对主克隆的非显著清除定义为治疗后VAF较治疗前下降<50％；如患者具有多个主克隆，所有主克隆VAF均下降≥50％，被视为显著清除，否则为非显著清除。

8. 随访：随访截止时间为2024年6月30日，采用住院病历、电话或门诊联系的方式随访。有效随访60例，失访9例，中位随访时间为22（5～72）个月。除5例（7.3％）行allo-HSCT的患者外，存活的患者有15例（21.8％），死亡的患者有40例（58.0％）。

9. 统计学处理：采用SPSS 27.0软件进行统计学分析，*P*<0.05为差异具有统计学意义。计数资料采用例数（百分比）形式描述，组间比较采用卡方检验或Fisher确切概率法；计量资料采用中位数（范围）描述，组间比较采用独立样本*t*检验（符合正态分布）或Mann-Whitney *U*检验（不符合正态分布）。总生存（Overall survival, OS）为疾病确诊时间至因任何原因死亡或末次随访时间，移植患者的OS为确诊时间至移植时间。采用Kaplan-Meier法绘制生存曲线，组间比较采用Log-rank检验。应用GraphPad Prism 8.0.2及R 4.4.1制图。

## 结果

一、患者临床特征

如表1所示，治疗前AZA组（24例）和DAC组（45例）相比，性别、年龄、HGB、PLT、WHO2016诊断分型、WHO2022诊断分型、IPSS-R预后分组、IPSS-M预后分组及治疗疗程数之间差异均无统计学意义，仅WBC（*P*＝0.012）和ANC（*P*＝0.014）组间差异有统计学意义。确诊MDS到启动HMA中位时间为1（0～25）个月。69例患者中，突变频率排前10位基因分别为TP53（15例，22％）、ASXL1（14例，20％）、RUNX1（13例，19％）、U2AF1（12例，18％）、DDX41（9例，13％）、SF3B1（9例，13％）、BCOR（8例，12％）、KMT2D（8例，12％）、STAG2（8例，12％）、TET2（7例，10％）（图1）。

**表1:** 69例骨髓增生异常综合征（MDS）患者接受去甲基化药物（HMA）治疗前的临床特征

临床特征	AZA组（24例）	DAC组（45例）	*P*值
年龄［岁，*M*（范围）］	62.5（46～74）	62（41～80）	0.730
性别［例（％）］			0.646
男	15（62.5）	32（71.1）	
女	9（37.5）	13（28.9）	
WHO 2016诊断分型［例（％）］			0.324
MDS-EB1	16（66.7）	23（51.1）	
MDS-EB2	8（33.3）	22（48.9）	
WHO 2022诊断分型［例（％）］			0.257
MDS-biTP53	1（4.2）	7（15.6）	
MDS-f	2（8.3）	1（2.2）	
MDS-IB1	14（58.3）	20（44.4）	
MDS-IB2	7（29.2）	17（37.8）	
WBC［×10^9^/L，*M*（范围）］	1.68（1.06～5.23）	2.18（0.88～13.60）	0.012
ANC［×10^9^/L，*M*（范围）］	0.62（0.14～3.62）	0.90（0.16～10.30）	0.014
HGB［g/L，*M*（范围）］	88（62～114）	79（43～138）	0.245
PLT［×10^9^/L，*M*（范围）］	47（15～188）	63（6～405）	0.207
IPSS-R预后分组^a^［例（％）］			0.910
中危	5（26.3）	13（31.7）	
高危	9（47.4）	18（43.9）	
极高危	5（26.3）	10（24.4）	
IPSS-M预后分组^a^［例（％）］			0.293
低危	0（0.0）	3（7.3）	
中低危	4（21.1）	3（7.3）	
中高危	3（15.8）	3（7.3）	
高危	6（31.6）	14（34.1）	
极高危	6（31.6）	18（43.9）	
HMA疗程［个，*M*（范围）］	7（4～23）	6（4～35）	0.446

**注** AZA：阿扎胞苷；DAC：地西他滨；MDS-EB：MDS伴原始细胞增多；MDS-biTP53：MDS伴TP53双等位基因改变；MDS-f：MDS伴纤维化；MDS-IB：MDS伴原始细胞增多；IPSS-R：修订的国际预后积分系统；IPSS-M：含分子遗传学指标的国际预后积分系统。^a^9例患者治疗前无可供分析的染色体核型，无法行IPSS-R和IPSS-M预后分组

**图1:**
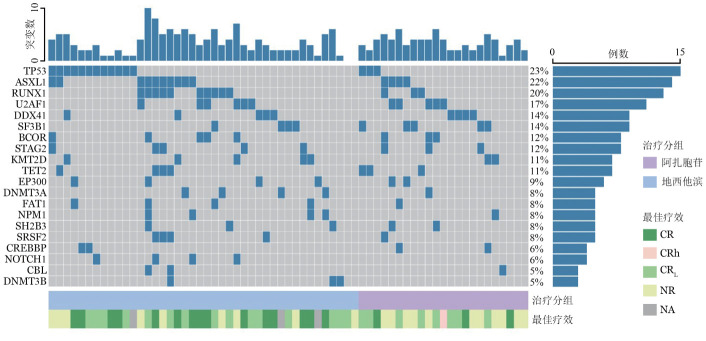
69例接受去甲基化药物治疗的骨髓增生异常综合征患者治疗前基因突变情况、治疗分组及4个疗程内最佳疗效 CR：完全缓解；CRh：完全缓解伴部分血液学改善；CR_L_：完全缓解伴有限血液学改善；NR：无效；NA：未评估疗效

二、疗效

AZA组24例患者中，CR 4例（16.7％），PR 1例（4.2％），CR_L_ 9例（37.5％）［其中CR_uni_ 5例（20.8％）、CR_bi_ 4例（16.7％）］，CRh 1例（4.2％），单纯HI 1例（4.2％），NR 8例（33.8％）；DAC组45例患者中，CR 17例（37.8％），CR_L_ 18例（40.0％）［其中CR_uni_ 7例（15.6％）、CR_bi_ 11例（24.4％）］，CRh 1例（2.2％），单纯HI 1例（2.2％），NR 8例（17.8％）。53例有效患者中，23例（43.4％）治疗1个疗程后起效，11例（20.8％）治疗2个疗程后起效，5例（9.4％）治疗3个疗程后起效，14例（26.4％）治疗4个疗程后起效。第1个疗程达最佳疗效10例（18.9％），第2个疗程达最佳疗效10例（18.9％），第3个疗程达最佳疗效7例（13.2％），第4个疗程后达最佳疗效26例（49.0％）。66例患者（AZA组24例，DAC组42例）在4个疗程内进行了疗效评估。AZA组中，4个疗程内最佳疗效为CR 3例（12.5％），CR_L_ 9例（37.5％）（CR_uni_ 6例、CR_bi_ 3例），CRh 1例（4.2％），NR 11例（45.8％）。DAC组中，CR 15例（35.7％），CR_L_ 17例（40.5％，CR_uni_ 12例、CR_bi_ 5例），CRh 1例（2.4％），NR 9例（21.4％）。IPSS-R预后分组和IPSS-M预后分组间的4个疗程内最佳疗效差异无统计学意义（*P*值分别为0.326和0.059）。治疗前患者伴或不伴有DNMT3A、TET2或ASXL1突变（DTA突变），信号转导基因突变（NRAS、KRAS、KIT、FLT3、CBL、MPL、NF1、JAK2），转录因子突变（RUNX1、ETV6、CUX1、GATA2），剪接因子突变（SF3B1、U2AF1、SRSF2、ZRSR2），TP53突变与4个疗程内最佳疗效差异均无统计学意义（均*P*>0.05）。

三、疗效持续时间

如表2所示，AZA组和DAC组患者组间DOR差异无统计学意义（*P*＝0.990）。LR-MDS和HR-MDS之间的DOR差异亦无统计学意义（*P*＝0.960）。治疗前伴或不伴有DTA突变、信号转导基因突变、转录因子突变、剪接因子突变患者之间的DOR差异均无统计学意义（均*P*>0.05）。将TP53突变进一步分为双打击（biTP53）和单打击（monoTP53）。bi TP53、mono TP53和不伴TP53突变患者中位DOR分别为3（95％*CI*：1～10）、10（95％*CI*：3～34）和16（95％*CI*：8～27）个月，三组间差异有统计学意义（*P*＝0.032）。

**表2:** 接受去甲基化药物治疗的骨髓增生异常综合征患者的中位疗效持续时间（DOR）和总生存（OS）时间比较［月，*M*（95％*CI*）］

组别	DOR		OS	
	时间	*P*值	时间	*P*值
治疗分组		0.990		0.883
AZA组	10（5～21）		31（15～62）	
DAC组	10（5～21）		32（16～65）	
IPSS-R预后分组		0.310		0.039
中危	9（2～9）		43（19～96）	
高危	19（4～19）		32（14～73）	
极高危	8（3～20）		25（11～55）	
IPSS-M预后分组		0.960		0.275
较低危	9（15～16）		46（11～28）	
较高危	16（4～71）		30（18～49）	
DTA突变		0.860		0.560
有	16（8～34）		35（17～73）	
无	9（4～18）		29（14～61）	
信号转导基因突变		0.106		0.087
有	55（10～NA）		57（9～97）	
无	9（6～21）		30（17～184）	
转录因子突变		0.363		0.764
有	26（6～21）		20（15～69）	
无	9（1～7）		32（9～42）	
剪接因子突变		0.201		0.433
有	16（8～32）		31（16～58）	
无	9（4～18）		33（17～61）	
TP53突变		0.032		<0.001
biTP53	3（1～10）		16（7～38）	
monoTP53	10（3～34）		15（6～40）	
无	16（8～27）		35（14～91）	

**注** AZA：阿扎胞苷；DAC：地西他滨；IPSS-R：修订的国际预后积分系统；IPSS-M：含分子遗传学指标的国际预后积分系统；DTA突变：DNMT3A、TET2或ASXL1突变；biTP53：双打击TP53突变；monoTP53：单打击TP53突变；NA：不可用

四、生存分析

1. 治疗前分子遗传学异常和预后分组与生存：如表2所示，AZA组和DAC组患者OS时间差异无统计学意义（*P*＝0.883）。IPSS-R预后分组为中危、高危和极高危组中位OS时间分别为43（95％*CI*：19～96）、32（95％*CI*：14～73）和25（95％*CI*：11～55）个月，组间差异有统计学意义（*P*＝0.039）。LR-MDS和HR-MDS组间OS时间差异无统计学意义（*P*＝0.275）。

治疗前伴或不伴有DTA突变、信号转导基因突变、转录因子突变、剪接因子突变患者之间的中位OS时间差异均无统计学意义（均*P*>0.05）。biTP53、monoTP53和不伴TP53突变患者中位OS时间依次为16（95％*CI*：7～38）、15（95％*CI*：6～40）和35（95％*CI*：14～91）个月，差异具有统计学意义（*P*<0.001）（表2）。

2. HMA治疗反应与生存：根据患者在4个疗程内获得的最佳治疗反应，将其分为CR组（18例）、CR_L_组（26例）、NR组（20例）（CRh组2例患者，因未达数据描述条件，未纳入分析），组间OS差异无统计学意义（*P*＝0.580）。CR组患者1、2、3年OS率分别为（94.1±5.7）％、（81.9±9.5）％、（54.6±12.8）％。CR_L_组1、2、3年OS率分别为（87.7±6.7）％、（60.8±10.3）％、（36.8±10.5）％。NR组1、2、3年OS率分别为100.0％、（49.2±14.8）％、（29.5±14.0）％（表3）。

**表3:** 骨髓增生异常综合征（MDS）患者接受去甲基化药物治疗4个疗程内最佳疗效分组的总生存（OS）分析

组别	中位OS时间（月，95％*CI*）	*P*值	1年OS率（％）	2年OS率（％）	3年OS率（％）
IWG 2023疗效分组		0.580			
CR组（18例）	41（19～89）		94.1±5.7	81.9±9.5	54.6±12.8
CR_L_组（26例）	31（11～50）		87.7±6.7	60.8±10.3	36.8±10.5
NR组（20例）	21（4～24）		100.0	49.2±14.8	29.5±14.0
主克隆清除程度分组		0.130			
主克隆显著清除（18例）	43（11～90）		94.4±5.4	94.4±5.4	59.4±13.0
主克隆非显著清除（12例）	31（15～127）		100.0	62.5±17.1	41.7±20.5
主克隆清除程度分组（非TP53突变）		0.013			
主克隆显著清除（14例）	55（9～106）		100.0	100.0	70.7±14.3
主克隆非显著清除（10例）	31（16～184）		100.0	57.1±18.7	0.0
IWG 2023疗效+主克隆清除程度分组		0.027			
获得疗效+显著清除（12例）	55（17～178）		91.7±8.0	91.7±8.0	71.3±14.1
NR/PD+显著清除（6例）	32（11～156）		100.0	100.0	40.0±21.9
NR/PD+非显著清除（10例）	25（5～72）		100.0	50.0±20.4	25.0±20.4
IWG 2023疗效+主克隆清除程度分组（非TP53突变）		<0.001			
获得疗效+显著清除（11例）	57（15～211）		100.0	100.0	77.8±13.9
NR/PD+非显著清除（8例）	19（5～71）		100.0	40.0±21.9	0.0

**注** IWG：MDS国际工作组；CR：完全缓解；CR_L_：完全缓解伴有限血液学改善；NR：无效；PD：疾病进展

截至随访终点，46例患者治疗后接受了NGS基因突变检测，其中30例（65％）伴有主克隆基因突变。根据HMA对于主克隆的清除情况，分为显著清除组（18例，60.0％）和非显著清除组（12例，40.0％）。HMA对于不同主克隆突变的清除效率不尽相同，高频突变中HMA对于U2AF1和ASXL1的清除较差（图2A）。在18例显著清除组中，6例（33％）为CR，6例CR_L_（33％）（CR_uni_ 3例、CR_bi_ 3例），5例（28％）NR，1例（6％）PD。在12例非显著清除组中，1例（8％）为CR_uni_，1例（8％）CRh，7例（58％）NR，3例（25％）PD。主克隆显著清除组与主克隆非显著清除组中位OS时间分别为43（95％*CI*：11～90）个月和31（95％*CI*：15～127）个月，差异无统计学意义（*P*＝0.130）（图2B）。但是，在非TP53突变患者中，主克隆显著清除组（14例）和主克隆非显著清除组（10例）中位OS时间差异有统计学意义［55（95％*CI*：9～106）个月对31（95％*CI*：16～184）个月，*P*＝0.013］（图2C）。其中，4个疗程内即表现为主克隆显著清除者（5例）和主克隆非显著清除者（10例）中位OS时间分别为43（95％*CI*：5～80）个月和35（95％*CI*：13～57）个月，主克显著清除组的3年OS率为100.0％，而主克隆非显著清除组仅为（41.7±20.4）％。获得疗效且主克隆显著清除的患者，3年OS率为（71.3±14.1）％，非TP53突变者3年OS率为（77.8±13.9）％，显著高于其他亚组（表3）。

**图2:**
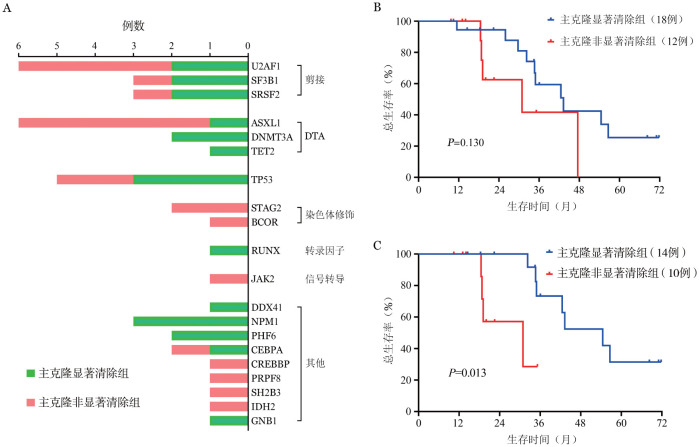
骨髓增生异常综合征伴原始细胞增多患者主克隆突变清除程度与生存 **A** 去甲基化药物对不同主克隆突变的清除程度；**B** 所有患者不同主克隆清除程度组生存曲线比较；**C** 非TP53突变患者不同主克隆清除程度组生存曲线比较

## 讨论

近年来，多项以HMA为基础的联合方案治疗HR-MDS的临床试验均宣告失败。因此，HMA单药仍然是HR-MDS的一线治疗选择。如何预测HMA的疗效及其对生存的获益仍然是一个重大的临床挑战。尽早确定无法从HMA治疗获益的患者，可以减少不必要的多疗程治疗和相应的不良反应，同时可尽早使患者直接行allo-HSCT或参加可能获益的临床试验。

两项Ⅲ期随机对照临床试验[12]–[13]均证实了AZA较常规治疗方案（包括最佳支持治疗、小剂量阿糖胞苷和急性髓系白血病样化疗）可显著提高HR-MDS的缓解率（中位疗程数：9，CR率：17％）并显著延长生存时间，与本研究中AZA组疗效相近（中位疗程数：7，CR率：16.7％）。多项Ⅲ期随机临床试验[14]–[16]也均证实DAC较支持治疗可显著提高HR-MDS的缓解率（中位疗程数：3，CR率：9％），延缓向急性白血病进展，有效者生存期显著延长。本研究中DAC组的CR率为37.8％（中位疗程数：6），与Kantarjian等[17]报道的真实世界中DAC标准剂量5天方案（中位疗程数：6）CR率可达39.0％相一致。因此，本研究中DAC组有效率较高，可能与入组患者均为接受至少4个疗程治疗者有关。

既往文献[8],[18]报道IPSS-R和IPSS-M预后积分系统均不能预测接受HMA治疗的MDS-EB患者的最佳疗效和DOR。我们的研究也发现IPSS-R预后分组不能预测接受HMA的疗效，但是IPSS-M预后分组的4个疗程内最佳疗效差异（*P*＝0.059）处于临界值，有待更大样本量的临床研究加以验证。既往Welch等[19]发现TP53突变、Bejar等[20]发现TET2突变可以预测HMA疗效，但亦有相关研究并未得出一致的结论[20]–[23]。我们的研究也提示治疗前是否伴有DTA突变、信号转导基因突变、转录因子突变、剪接因子突变与最佳疗效、DOR和OS差异均无统计学意义。治疗前TP53突变与最佳疗效差异无统计学意义，但是TP53突变状态与DOR相关，伴有bi TP53或monoTP53突变患者的DOR较不伴TP53突变者显著缩短，这也解释了接受HMA治疗的TP53突变患者最佳疗效差异无统计学意义但是生存期极短的原因。我们分析了不同最佳疗效患者的OS，虽然各疗效分组间差异无统计学意义，但是从2年和3年的生存率来看，获得深度缓解的患者有望获得长期且持续的疗效。

MDS-EB患者的中位基因突变数目为2～3个[24]–[25]。MDS克隆对于包括HMA敏感性在内的生物学行为可能由不同的基因突变组合所决定，而非单个基因突变所决定。Nazha等[23]基于人工智能算法提出了多个包含ASXL1突变的突变组合与HMA耐药相关。我们的研究发现治疗前除TP53突变外，其他单一基因突变，甚至纳入了多种分子遗传学不良预后因素的IPSS-M积分均不能预测患者接受HMA的疗效。在本研究队列中，60％患者伴有主克隆突变，而主克隆突变被认为是驱动MDS发生的重要因素[25]–[26]。我们的结果提示HMA对于主克隆清除的程度与治疗反应和OS密切相关。非TP53突变患者中，HMA治疗有效且主克隆显著清除者的3年OS率高达77.8％。

综上所述，我们认为通过NGS基因突变检测，评估治疗后HMA对主克隆的清除程度较治疗前的基因突变状态更有助于预测患者的长期疗效和生存。主克隆清除不显著者应尽早接受allo-HSCT、二线治疗或参与可能获益的临床试验。本研究存在以下不足之处：样本数有限且为回顾性分析，随访时间尚短。因此，未来仍需更大样本量前瞻性临床试验来进一步验证上述发现。
